# Relational interventions for organizational learning: An experience report

**DOI:** 10.1002/lrh2.10270

**Published:** 2021-05-02

**Authors:** N. Marcus Thygeson, Caroline Logan, Curt Lindberg, Jennifer Potts, Anthony Suchman, Robert Merchant, Randy Thompson

**Affiliations:** ^1^ Adaptive Health San Rafael CA USA; ^2^ ABT Associates Inc Cambridge MA USA; ^3^ Partners in Complexity Waitsfield VT USA; ^4^ Billings Clinic USA; ^5^ Relationship Centered Health Care University of Rochester School of Medicine and Dentistry Rochester New York USA

**Keywords:** learning organizations, process improvement, relational coordination, self‐determination theories, quality improvement

## Abstract

**Introduction:**

Quality improvement and implementation science practitioners identify relational issues as important obstacles to success. Relational interventions may be important for successful performance improvement and fostering Learning Health Systems.

**Methods:**

This case report describes the experience and lessons learned from implementing a relational approach to organizational change, informed by Relational Coordination Theory, in a health system. Structured interviews were used to obtain qualitative participant feedback. Relational Coordination was measured serially using a validated seven‐item survey.

**Results:**

An initial, relational intervention on one unit promoted increased participant engagement, self‐efficacy, and motivation that led to the spontaneous, emergent dissemination of relational change, and learning into other parts of the health system. Staff involved in the intervention reported increased systems thinking, enhanced focus on communication and relationships as key drivers for improvement and learning, and greater awareness of organizational change as something co‐created by staff and executives.

**Conclusions:**

This experience supports the hypothesis that relational interventions are important for fostering the development of Learning Health Systems.

## 1 INTRODUCTION

Quality improvement practitioners report a low overall success rate for their projects, a trend supported by the literature from the past 20 years.^1^, ^2^, ^3^, ^4^ These same practitioners identify relational rather than technical issues as the principal obstacles and report that their current tools and approaches do not adequately prepare them for these relational challenges.^1^, ^5^ Implementation science practitioners also identify relational factors as key contributors to successful innovation^6^, ^7^; and workforce engagement and knowledge sharing have been identified as critical elements of learning organizations.^8^ The literature on Learning Health Systems is focused on several key themes, especially the technical infrastructure (electronic health records and the like) required to acquire, store, and analyze clinical data and to produce new knowledge for improving clinical performance.^9^ In contrast, the social infrastructure of Learning Health Systems does not appear to have received as much attention,^10^ despite the fact that most health care is delivered by interprofessional teams and networks of collaborating providers. Information and knowledge flow between individuals and teams via a social network. Social networks strongly influence organizational performance, and are likely to be another important domain for study and intervention in Learning Health Systems (LHSs).^11^ New approaches to addressing the relational dimensions of process improvement are needed to accelerate the development of LHSs.

Relational Coordination Theory (RC) is an approach to exploring team collaboration and performance that shares key concepts with complexity science. RC describes seven dimensions of interaction that allow collaborating individuals, workgroups, and/or organizations to coordinate their work and actively manage the interdependencies of their tasks.^12^ Four dimensions describe characteristics of communication: frequency, timeliness, accuracy, and when a problem arises, a focus on solving the problem rather than assigning or deflecting blame. The other three dimensions are qualities of relationship: shared goals for the work process, shared knowledge of each other's work, and mutual respect for each other's work (Gittell 2006)^13^ RC can be measured by a validated survey, with the resulting score indicating the strength of the network of ties among the collaborating workgroups.^14^, ^15^

Extensive research shows that higher levels of RC are consistently associated with higher levels of performance, including clinical outcomes, safety, cost, patient experience, staff satisfaction and well‐being, and the capacity to innovate (e.g.,^14^, ^16^, ^17^, ^18^, ^19^, ^20^, ^21^, ^22^, ^23^, ^24^, ^25^, ^26^, ^27^).

This experience report describes one of the first applications of Relational Coordination Theory as a framework for process improvement, organizational learning, and change management. RC focuses on improving communication, relationships, alignment, and systems awareness to foster more effective collaboration on interdependent tasks, ultimately resulting in performance improvement. We found that a single, relatively simple intervention on one unit promoted a high degree of engagement, learning, improved self‐efficacy, and motivation among the participants that led to an unexpected, emergent cascade of dissemination across the health system.

### 1.1 Questions of Interest:

1. Is RC an effective strategy for promoting learning and change management in a complex care delivery system?
2. What is the impact of an RC intervention on the participants in that intervention?
3. What challenges do health care workers experience implementing RC?

## 2 METHODS

### 2.1 Organizational context

This project was conducted in a regional healthcare system, anchored by a multi‐specialty group practice, a tertiary care hospital, and a network of critical access hospitals. The system, which serves a rural population in a sparsely populated part of the United States, is physician‐led and recognized for its culture of innovation and commitment to learning.^28^ Clinical departments are led by physician‐manager dyads. Novel improvement methods and cultural change frameworks are routinely tried and evaluated. A well‐developed Operational Excellence department focuses on operational efficiencies using methods from lean and Six Sigma. In addition, organizational leaders and staff, including the Chief Medical Officer (CMO) and the Director of the Partnership for Complex Systems and Healthcare Innovation (partnership director) participated in a self‐study group focused on using complexity science and related change processes, such as RC and positive deviance, in healthcare. Systems science is an essential and distinguishing attribute of LHS research identified by the Agency for Healthcare Research and Quality.^29^ Complexity science, as applied to organizations, focuses attention on the emergent, self‐organizing nature of human interaction, and calls for a leadership approach that emphasizes process reflection, relational quality, and co‐creation, with less emphasis on command and control strategies.^30^

Intrigued by the RC approach to system‐ness and collaboration, the complexity study group began searching for an opportunity to trial an improvement project informed by RC. The project began in 2012 and is ongoing. We report interim results as of 2017.

The first opportunity to apply RC was the relocation of the intensive care unit (ICU). The ICU staff was anticipating a move to a new facility that featured a physical footprint almost three times larger than the old one and multiple nursing pods rather than one large central workspace. The staff was concerned that the spatial separation would negatively impact their ability to communicate and collaborate, and thus, the quality of patient care.

### 2.2 The RC intervention

The RC intervention was implemented iteratively in successive waves, introducing the concepts of interdependence and RC, measuring the RC between various workgroups, discussing the results and lessons learned, and taking action based on the findings. Because of the iterative learning nature of the intervention, we report RC survey results in the Methods section.

The first step involved outreach and engagement on the part of the CMO and the Partnership Director. They circulated articles about RC to the ICU staff and attended meetings of various groups (eg, the ICU nursing partnership council, department of pulmonary‐critical care medicine, and the rehabilitation department) to present an overview of RC and to ask whether it made sense to these groups to employ RC as a framework to enhance collaboration in the face of the impending move. The response from front‐line staff, pulmonary‐critical care physicians, and ICU leadership was widespread agreement. The ICU staff was known for valuing collaboration and a willingness to try innovative improvement approaches.

During these various meetings and related informal conversations, the CMO and the Partnership Director identified staff members who seemed particularly enthusiastic about this approach and invited them to join an interprofessional project team to plan and lead the effort.

Many of them possessed an existing interest in multidisciplinary collaboration. The initial project team included two occupational therapists, two intensivists, the ICU case manager, a physical therapist, nurse, critical care pharmacist, ICU clinical coordinator, speech therapist, and respiratory therapist. The case manager, one of the occupational therapists, and the clinical coordinator emerged as leaders by stepping forward to communicate actively with ICU colleagues, build interest, and pilot changes. The CMO and the Partnership Director liaised with system leadership and provided consultative support on implementation science, staff engagement strategies, and relational coordination. The CMO was also an actively practicing pulmonary‐critical care physician. He thus brought first‐hand knowledge about ICU operations and direct senior leadership support for the project.

The project team, which named itself “ICU Connections”, then conducted a survey administered by Relational Coordination Analytics to measure baseline levels of relational coordination among the ICU staff. They identified the core workgroups involved in ICU care ‐ nurses, nurse managers, care managers, intensivists, rehabilitation therapists, dietitians, and respiratory therapists ‐ that would complete the survey about their own workgroup and each of the other workgroups. In all, 112 staff members were surveyed and 79 responded for a response rate of 71%. To gain further insights into collaboration in the ICU, the team decided to query patients, or family members when a patient's condition was such that they could not complete the survey. Sixty‐three patient and family surveys were completed; considered a representative sample. Waivers of patient consent and privacy authorization were obtained from the Billings Institutional Review Board prior to collecting patient and family surveys.

**FIGURE 1:**
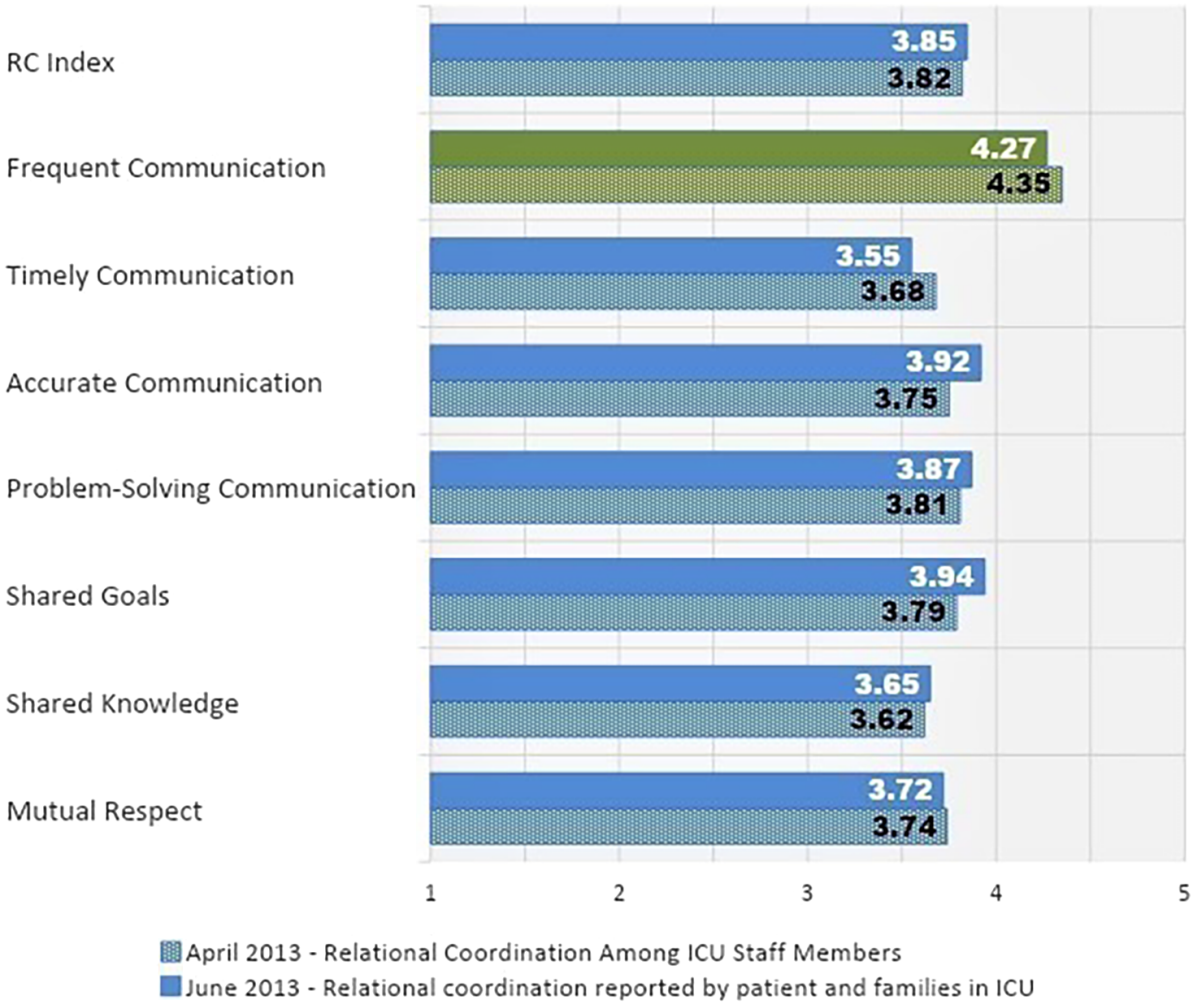
Results of the baseline Relational Coordination surveys showing ratings of the ICU staff by ICU staff members, including physicians, and by patients and family members. On a scale of 1 to 5 less than 3.5 is considered weak performance (orange bars), 3.5 to 4 moderate performance (blue bars), greater than 4 strong performance (green bars)

Figure 1 shows the ratings of the seven RC dimensions across all workgroups by staff and patients/family members. Interestingly, the ratings by patients/family members and staff were quite similar. The RC Index, a composite of ratings on all seven dimensions measured on a five‐point scale, was 3.82 as reported by staff and 3.85 as reported by patients/family members. Frequency of communication was the most highly rated dimension by both groups while timely communication and shared knowledge were the two lowest.

**FIGURE 2:**
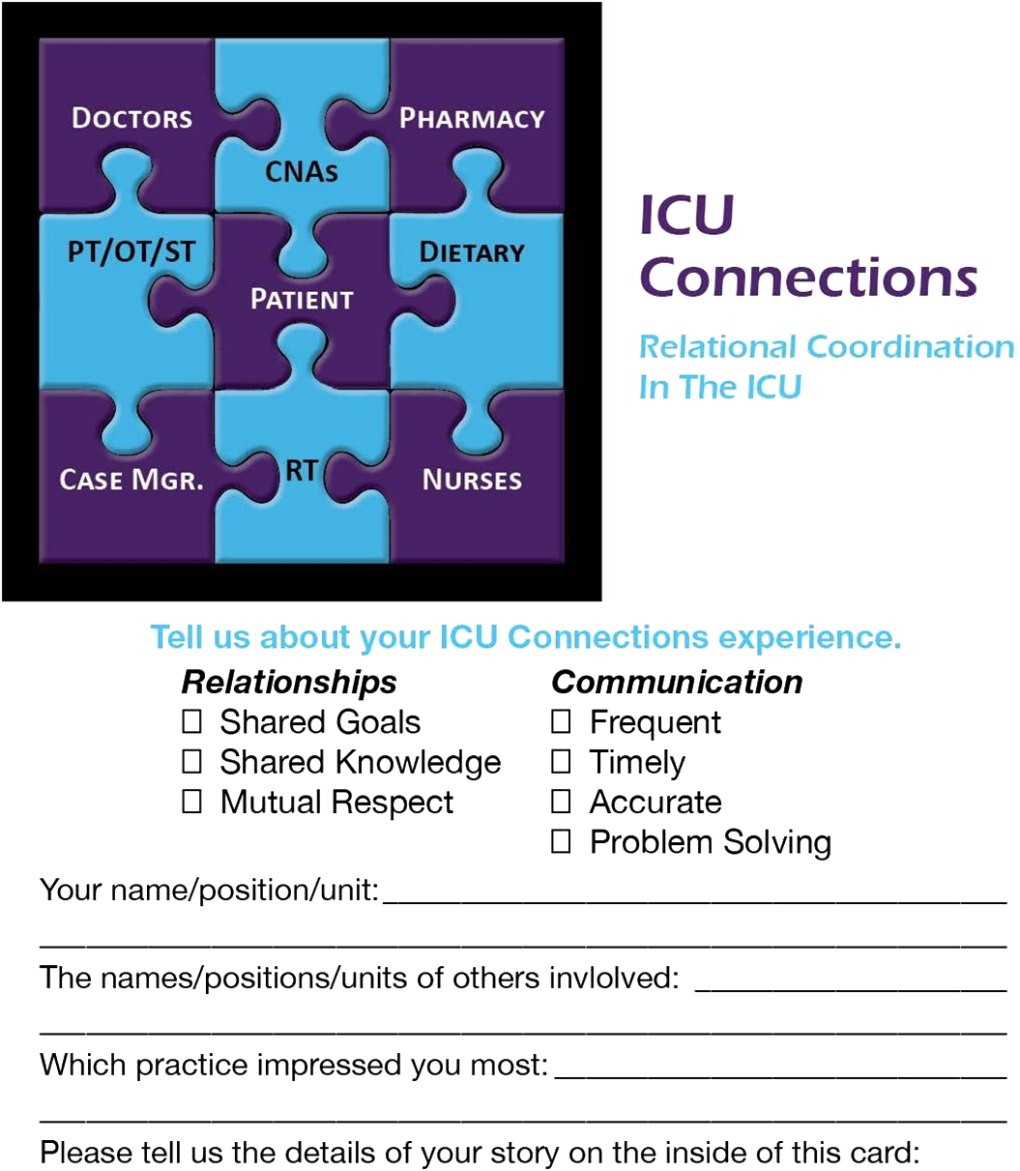
Example of “RC Bingo” intervention

Responding to patterns revealed by the survey, the project team created two initial interventions ‐ a newsletter and RC Bingo ‐ to help the various workgroups in the ICU better understand and thus be more responsive to each other's work. The latter was a game in which staff members nominated peers from professions other than their own whose actions supported relational coordination (see Figure 2). Nominations were posted on a Bingo Board with workgroups on one axis and the RC dimensions on the other. The first profession to receive nominations on all seven RC dimensions was awarded a pizza party.

Motivated by their workgroup's initial low score of 3.33 on the RC survey, the occupational and physical therapists on the project team initiated two activities to improve working relationships with their ICU colleagues. They started contacting nurses each morning to discuss their patients' care plans, particularly their needs for therapy, and the optimal timing for therapy. They also conducted a chart review to determine what they could have added to the care of ICU patients for whom they had not been consulted. They shared the findings of the chart review and care plan ideas with ICU physicians and nurses and on daily rounds to increase the staff's knowledge of the capabilities and potential impact of the therapists. In the year following these activities, the proportion of ICU patients receiving OT and PT therapy rose from 28% to 70%.

When a chaplain heard about the RC survey, she asked why chaplains had not been included. The project team immediately invited her to join ICU Connections and committed to including chaplains in the next survey. The chaplain soon identified another communication issue: the lack of timely notification of chaplains when patients were placed on “comfort care.” Typically, they only learned of such orders when they spotted a cart of refreshments for family members outside a patient's room. This triggered an invitation to the ICU nurse informaticist to join the project team. Within days, the nurse informaticist created an automatic paging system to alert chaplains as soon as a “comfort care” order was written. Over the ensuing year, the average number of end‐of‐life ministry consultations in the ICU rose from 4 to 28 per month.

The experience with therapists and chaplains led the project team to adopt an expanded view of who was on the ICU team, by thinking more systemically. One result of this was an ICU RC Summit, held in late 2013, to which all ICU personnel was invited. Over sixty professionals attended to learn about RC, analyze survey results, hear from peers about their efforts to foster collaboration, and plan the next phase of the RC initiative. This resulted in plans for various improvements in interprofessional rounding and new uses of the electronic health record to share information allowing better coordination of tasks. Changes led by front‐line staff over the beginning of 2014 included family participation in daily rounds, chaplains partnering with family members on daily rounds, more all‐staff RC workshops; and a new, easily accessible electronic health record page to share goals of care, daily plans, and other critical information with the whole team. The strategies to better engage patients and family members were informed by the RC survey findings.

**FIGURE 3:**
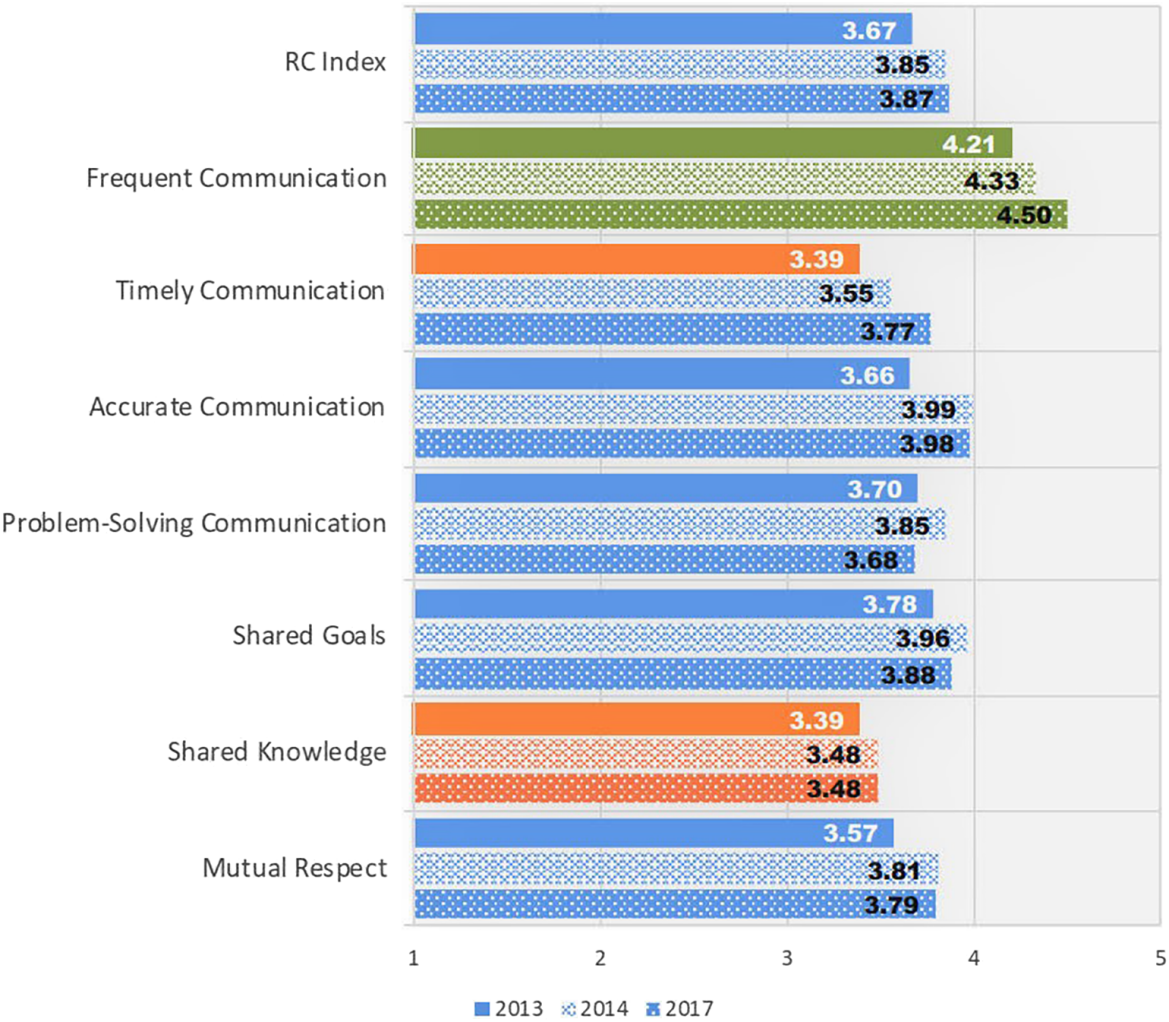
Comparison of RC Surveys showing ratings of the ICU Staff by core ICU staff members (nurses, nurse managers, care managers, pharmacists, dietary staff, speech pathologists, and respiratory and rehabilitation therapists) at baseline (2013), during the intervention (2014) and after the intervention (2017). Differences in how physicians' ratings were gathered and aggregated in the 3 time periods precluded a valid comparison, so their ratings have been excluded from this analysis. Based on field experience, but not statistical studies, changes of 0.1 points or more on the 5‐point scale are considered clinically significant (personal communication, Jody Hoffer Gittell). On a scale of 1 to 5, less than 3.5 is considered weak performance (orange bars), 3.5 to 4 moderate performance (blue bars), greater than 4 strong performance (green bars)

**FIGURE 4:**
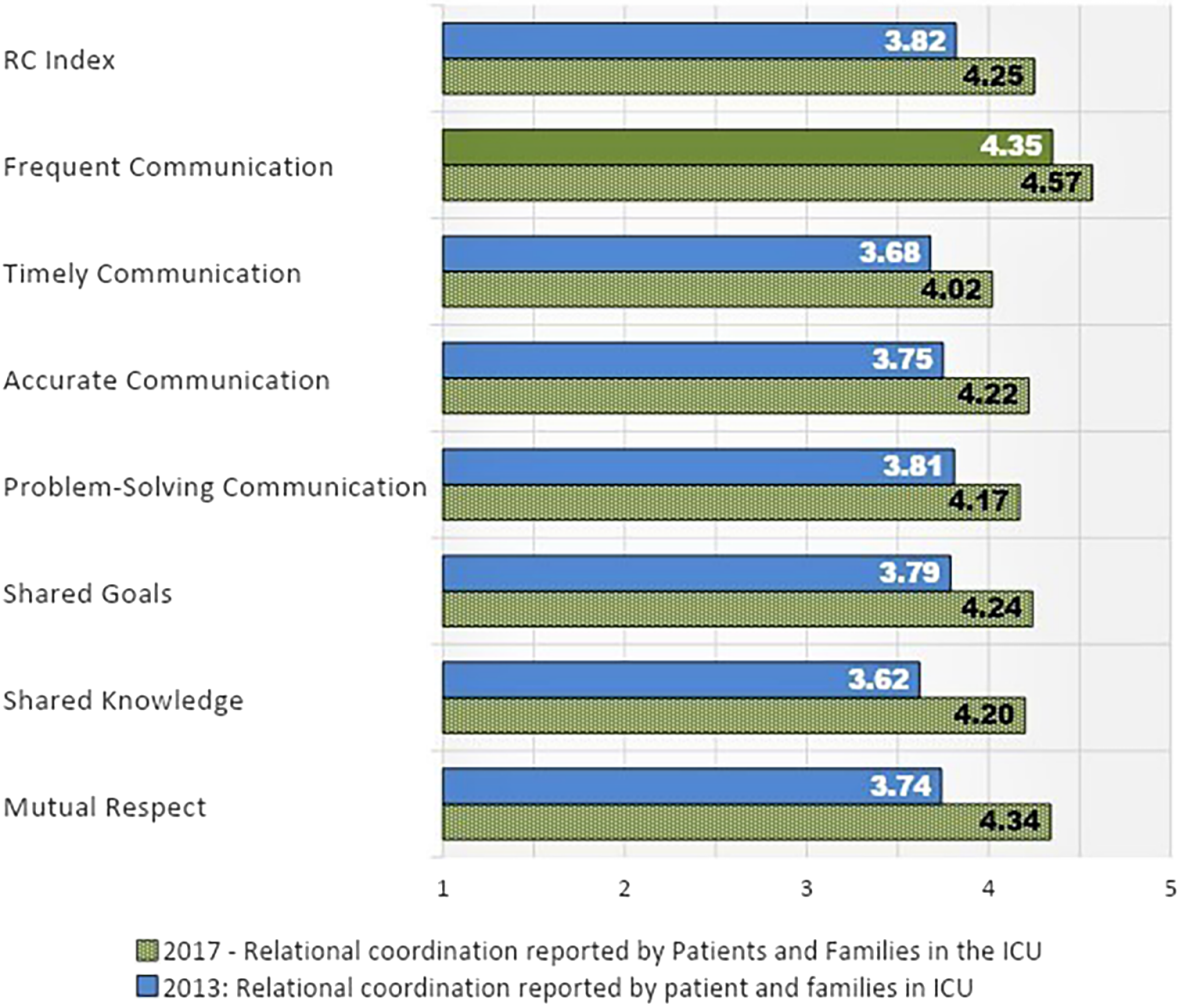
Relational coordination reported by Patients and Families in the ICU at baseline (2013) and after intervention (2017). Blue bars indicate “moderate” and green bars indicate “strong” RC performance

Follow‐up RC surveys of and by the ICU staff were conducted in 2014 and 2017 showing both initial and sustained improvement in overall RC and six of the seven dimensions (Figure 3). The workgroup with the largest improvement was physical and occupational therapy, whose RC Index score rose from 3.33 to 4.00. A second survey of patient/family members' rating of RC in the ICU staff was also conducted in 2017, showing substantial increases in all seven RC dimensions from the perspective of family and caregivers (Figure 4). The organization's annual employee engagement survey also showed improvement from 2014 to 2015. The proportion of ICU nursing staff who rated themselves as “engaged” increased from 11.5% to 23.5%. There was a heightened sense of collaboration despite the disruptions caused by the move to the new unit.

RC now is embedded in the culture of the ICU. Most recently, an interprofessional team has been implementing RC‐informed interventions to prevent delirium and decrease ventilator days.

As news of success in the ICU spread informally throughout the organization, other groups began to apply RC in their work. Several members of the ICU Connections project team joined with colleagues in other units to enhance teamwork between outpatient, inpatient, and homecare staff for patients undergoing joint replacement surgery. They called themselves the “Joint Ventures.” One year later, when the hospital opened a new orthopedic/neurology unit, “Joint Ventures” staff members formed an inter‐professional committee to create a collaborative learning culture on the new unit. Soon thereafter, RC theory was incorporated into the curriculum of the new Nurse Residency program. RC has rippled from the ICU to many other parts of the organization as staff members have brought their RC experience to new improvement opportunities. With each project, more staff members have gained RC skills and more informal RC leaders have emerged. Together these informal leaders have formed an RC Learning Network. In 2017, senior leaders integrated RC into the organization's new improvement methodology and established improved teamwork and relational coordination as a central organizational objective, thus embedding RC in the organization's culture.

### 2.3 Assessing the impact of the RC intervention on staff

After 5 years of implementing and spreading RC across the organization, we investigated the impact of RC on participating employees by conducting semi‐structured interviews with a purposive sample of twenty‐one staff members who were involved in one or more RC‐informed improvement efforts. Interviewees represented six professions ‐ nursing, rehabilitation, pharmacy, organizational development, medicine, and information technology ‐ and included frontline staff, middle managers, and senior executives. Interview responses were recorded and transcribed. We then conducted a formal content analysis in which each of the authors independently reviewed the interview responses identifying the key themes, reviewed each other's thematic categories, and engaged in iterative discussions until consensus was achieved. This employee interview process was determined to be exempt from Institutional Review Board approval by the Billings Institutional Review Board.

## 3 RESULTS

Qualitative analysis of the interview transcripts yielded four major themes, illustrated with some exemplar quotations from the survey.

### 3.1 RC changes how people think about their work

It fosters systems thinking, helping people understand their own work in the context of the larger whole. It helps staff pay more attention to who else is involved in their work processes, to value multiple perspectives, and to heighten awareness of interconnections and interdependencies.

*It's been eye opening ‐ how many people depend on each other to get things done… I no longer take for granted the work of others and am careful about making assumptions about what they do and why they do it*. (Case manager).

*The whole foundation of the interdisciplinary team does not just exist at the bedside, but at the leadership level and between units and departments. RC has helped me appreciate this and focus more on relationships and less on personalities*. (Unit manager).

### 3.2 RC promotes inclusiveness and improves collaboration

It increases relationship‐building, communication, and shared decision‐making, and decreases blaming. It encourages people to engage with and value the input of all participants in a work process.

*Before my exposure to RC I thought all aspects of care should be driven by nursing. Now I see it*'*s a team process. As a consequence, I*'*m much more inclusive now*. (ICU nurse).

*The comfort level of professionals from different disciplines in the ICU with raising concerns, making suggestions, and solving problems together has increased dramatically*. (Nursing director).

*In a meeting this morning with a neurosurgeon and nursing leaders about concerns the neurosurgeon had about quality on the new ortho‐neurosurgery unit and thinking about how to deal with these concerns, I could have issued a new policy or some instructions to staff on how to deal with the problems. In this case, I thought it best to focus on interactions and relationships between the neurosurgeons and nursing staff. I drew on key RC principles, like shared goals and shared knowledge, to facilitate what turned out to be a very productive exchange*. (Physician executive).

### 3.3 RC promotes personal development and fulfillment

The new perspectives, behavior changes, improved relationships, and improved performance associated with RC increased people's sense of self‐efficacy and confidence. RC fostered learning and increased the joy, meaning, and pride that people experience in their work.

*The experience in the ICU made me feel part of something important and helped me realize I can make contributions to the organization*. (Occupational therapist).

*I*'*m a new leader here and have had to learn a lot. RC has given me new perspectives and greater self‐awareness. The theory has also helped me learn to welcome different perspectives. It*'*s opened my eyes so much that I'll be including RC in one of my personal development goals for the coming year*. (Pharmacy manager).

*It makes the job better. Being friends and caring about the people you work with makes work more fun. It is especially helpful on the stressful days when all you do is run from emergency to emergency*. (Intensivist).

### 3.4 RC improves the organizational climate and strengthens employee engagement and commitment

*Seeing the passion of those involved in the ICU effort left a mark. I came to appreciate how much more colleagues can contribute if given the opportunity to work on something they care about and processes like RC to guide them*. (Nurse informaticist).

*[RC has] significantly impacted how I feel about [the organization] and my work… [it] has added spice to my work. It's also given me the opportunity to get to know people in other disciplines and in leadership. Together, this experience has given me great faith in the organization and its direction*. (Occupational therapist).

Interviews also identified two challenges staff experienced with the RC intervention. First, the novelty of RC induced initial skepticism from some colleagues, and there was discomfort with the terminology of RC, which some perceived as jargon. The project team accommodated this discomfort by using the term ICU Connections, rather than mentioning RC specifically. Subsequently, with familiarity, the language of RC became widely used throughout the organization. Secondly, the project team encountered minor, temporary resistance to some of their interventions, particularly family rounding, which was overcome by allowing physicians to choose whether to participate. Eventually, all chose to participate.

## 4 DISCUSSION

In contrast to most improvement efforts where a large effort often produces little or no benefit, we were struck that one modest RC‐based intervention to address one concern in one unit could lead to a cascade of initiative, engagement, and learning across many parts of the organization. This relationally focused intervention not only succeeded at its original tactical goal of maintaining cohesiveness among the ICU staff as they relocated to larger decentralized quarters, but also initiated a cascade of other process improvements in the ICU and elsewhere.

Qualitative feedback from survey participants indicates the RC intervention fostered learning at a number of levels in the organization. Staff learned to see their workplace more systemically; they acquired a more accurate and complete understanding of the work of their co‐workers; and they learned to value that work more highly. The RC intervention manifested several characteristics we associate with learning activities. First, participating staff acquired new perceptions, facts, and understanding, as listed above. Second, the spontaneous spread of the RC intervention through the organization, engaging new people on new projects, indicates it had the self‐reinforcing and positively attractive virtues of a learning activity.

Key success factors and takeaways are:

- The ability of the RC framework to foster a systems perspective in the ICU staff. RC made team interdependencies visible and discussable, prompting staff members to improve their communication and relationships, and carry out their work with greater mindfulness of the system and their impact on each other. Systems thinking is considered a key competency in LHSs.^29^ RC raised participant awareness about the social organization of their care team and resulted in changes in both the structure and function of that team.
- The diagnostic framework provided by the RC survey directed activity toward what mattered most to participants. For example, timely communication was the lowest rated RC dimensions, so the project team focused on it and not on respect, communication skills, or other areas that were not problematic. Without the survey, the team would only have been guessing about where to focus. Using near real‐time data about team performance to inform and improve performance is another key characteristic of LHSs.
- The RC approach emphasizes co‐creation. This helped the executives resist the temptation to impose a top‐down solution and instead engage the ICU staff as co‐designers of the intervention. They did introduce the first intervention, the RC survey, but only with the awareness and agreement of the ICU staff; after that the grass roots project team created every subsequent intervention. In accord with Self‐Determination Theory, a theory of intrinsic motivation and behavior change, this support for the staff members' autonomy enhanced their engagement, motivation and commitment, and learning as demonstrated in the interviews.^31^

Interventions like the one we have described are complex, and the success of such interventions is dependent on context, proper matching of the intervention to the “problem”, and expertise in execution. This RC‐informed initiative took place in an organizational setting characterized by pre‐existing familiarity and engagement with complexity science concepts, and strong leadership support for the application of these concepts for learning and improvement. While little is known about the configurations of conditions associated with success or failure of RC‐informed interventions, it seems plausible that the characteristics of the organization played an important role in enabling the outcomes we observed.

## 5 CONCLUSION

In summary, we have described our experience applying a relational approach to process improvement based on Relational Coordination Theory. This approach allowed the staff of an ICU to see for themselves the patterns of communication, relationship, and interdependence among the different workgroups and to design specific interventions for themselves. Their efforts succeeded well beyond the project's initial goal (maintaining staff cohesiveness and communication in a new physically‐dispersed facility) by spawning additional improvement initiatives, improving patients' and families' perceptions of staff collaboration with them, and having a positive impact on participants (fostering systems thinking, inclusiveness, collaboration, and engagement). Our experience suggests that relational interventions, by addressing the social infrastructure of health systems, are important for fostering the development of LHSs.

## CONFLICT OF INTEREST

The authors declare no conflicts of interest.
